# Which method of left atrium size quantification is the most accurate to recognize thromboembolic risk in patients with non-valvular atrial fibrillation?

**DOI:** 10.1186/1476-7120-12-28

**Published:** 2014-07-22

**Authors:** Ana Faustino, Rui Providência, Sérgio Barra, Luís Paiva, Joana Trigo, Ana Botelho, Marco Costa, Lino Gonçalves

**Affiliations:** 1 Cardiology Department, Coimbra’s Hospital and University Centre – General Hospital, Coimbra, Portugal; 2 Faculty of Medicine, University of Coimbra, Coimbra, Portugal

## Abstract

**Background:**

Left atrial (LA) size is a predictor of cardiovascular outcomes in patients in sinus rhythm, whereas conflicting results have been found in atrial fibrillation (AF). This study aims to: (1) Evaluate the accuracy of LA size to identify surrogate markers of an increased thromboembolic risk in patients with AF; (2) Assess the best method to evaluate LA size in this setting.

**Methods:**

Cross-sectional study enrolling 500 consecutive patients undergoing transthoracic and transesophageal echocardiography evaluation during a non-valvular AF episode. LA size was measured on transthoracic echocardiography using several methods: anteroposterior diameter, area in four-chamber view, and volumes by the ellipsoid, single- and biplane area-length formulas. Surrogate markers of stroke were evaluated by transesophageal echocardiography: LA appendage (LAA) thrombus, LAA low flow velocities, dense spontaneous echocardiographic contrast and LA abnormality.

**Results:**

Except for non-indexed anteroposterior diameter, increased LA size quantified by all the other methods showed a moderate to high discriminatory power to identify all the surrogate markers of stroke. A higher accuracy was observed for indexed LA area in four-chamber view (LAA thrombus: AUC = 0.708, CI_95%_ 0.644- 0.772, p<0.001; LAA low flow velocities: AUC = 0.733, CI_95%_ 0.674- 0.793, p<0.001; dense spontaneous echocardiographic contrast: AUC = 0.693, CI_95%_ 0.638- 0.748, p<0.001; LA abnormality: AUC = 0.705, CI_95%_ 0.654-0.755, p<0.001), indexed single-plane area-length volume (LAA thrombus: AUC = 0.701, CI_95%_ 0.633-0.770, p<0.001; LAA low flow velocities: AUC = 0.726, CI_95%_ 0.660-0.792, p<0.001; dense spontaneous echocardiographic contrast: AUC = 0.673, CI_95%_ 0.611-0.736, p<0.001; LA abnormality: AUC = 0.687, CI_95%_ 0.629-0.744, p<0.001), and indexed biplane area-length volume (LAA thrombus: AUC = 0.707, CI_95%_ 0.626-0.788, p<0.001; LAA low flow velocities: AUC = 0.737, CI_95%_ 0.664-0.810, p<0.001; dense spontaneous echocardiographic contrast: AUC = 0.651, CI_95%_ 0.578-0.724, p<0.001; LA abnormality: AUC = 0.683, CI_95%_ 0.617-0.749, p<0.001), without significant difference between them. Indexed LA area in four-chamber view and indexed area-length volumes also were independent predictors of surrogate markers of stroke.

**Conclusions:**

Left atrium enlargement is associated with an increased prevalence of surrogate markers of stroke in patients with non-valvular AF. Indexed LA area in four-chamber view and indexed area-length volumes displayed the strongest association.

## Introduction

Left atrium (LA) dilation has been associated with adverse cardiovascular outcomes in patients with sinus rhythm [1-5]. Among patients with atrial fibrillation (AF), LA enlargement has been suggested as predictor of stroke in some studies [6-11], while others have shown conflicting results [12].

Most investigations regarding the prognostic impact of LA size relied on the antero-posterior diameter measured by M-mode (LA AP diameter). However, this method is based in several geometric assumptions that often result in LA size underestimation [11-16]. Thus, LA areas and volumes derived from two-dimensional transthoracic echocardiography (TTE) seem to provide a more accurate assessment of LA size [17-19].

Transesophageal echocardiography (TEE) allows the assessment of parameters associated with stroke, thromboembolism and adverse prognosis, notably the presence of thrombus in the LA appendage (LAA thrombus) [20], spontaneous echocardiographic contrast (SEC) [20,21], low LA appendage flow velocities (low LAA velocities) [22-25]. The best method to assess the relationship between LA size and the aforementioned LA abnormalities on TEE has never been evaluated.

This study aims to evaluate the accuracy of LA size to identify surrogate TEE markers of an increased risk of stroke (LAA thrombus, low LAA velocities and dense SEC), and also to assess the best method to evaluate LA size in this setting.

## Methods

### Study design

Cross-sectional study enrolling patients undergoing TTE and TEE during a non-valvular AF episode. LA size was measured on TTE by several methods (LA AP diameter, LA area and LA volumes). Surrogate markers of stroke were evaluated by TEE: LAA thrombus, low LAA velocities, dense SEC, and LA ABN. ROC curve analysis was performed to assess the accuracy of the different LA measurements to identify surrogate markers of stroke. All patients provided informed consent for echocardiographic evaluation. The study protocol was approved by *Comissão de Ética da Faculdade de Medicina da Universidade de Coimbra* (Ref 5/2013 ).

### Patients and eligibility criteria

Five hundred consecutive adult patients undergoing TTE and TEE during symptomatic episode of non-valvular AF were enrolled during a 36-month period. AF was identified by an electrocardiogram or endocardial electrograms (in patients with pacemakers or implantable cardioverter defibrillators). Exclusion criteria were moderate or severe mitral stenosis, severe mitral regurgitation, severe aortic stenosis prosthetic mitral or aortic valves, patients with unsuitable images for accurate assessment of TTE measurements or TEE surrogate markers of stroke, or any contraindication to TEE. Most patients had an echocardiogram performed as part of evaluation before electrical cardioversion (n = 466, 93.2%); in the remaining patients, it was conducted for mitral valve disease assessment (n = 14; 2.8%) and stroke evaluation (n = 20; 4%).

### Initial data collection

Demographic, anthropometric, clinical, laboratory, additional echocardiographic and medication data were retrieved from clinical records (outpatient clinic evaluations, emergency department, and hospital-ward admissions). Discharge primary and secondary diagnoses were recorded according to the ICD-10 (international classification of diseases, 10th revision). Duration of AF episode was estimated according to the patients’ complaints, previously available clinical records and ECG. In patients with pacemakers and implantable cardioverter defibrillators device interrogation was performed in order to estimate the duration of the episode. The CHADS_2_[26] and CHA_2_DS_2_-VASc [27] scores, glomerular filtration rate (MDRD formula), body mass index and body surface area were calculated for all participants. Laboratory tests were performed within 24 hours of hospital admission for patients undergoing TEE in the emergency department and in the month preceding the transesophageal echocardiographic examination for participants undergoing elective TEE. Patients were thought to have congestive heart failure whenever concordant signs or symptoms were registered in their medical records. Vascular disease was defined by the presence of at least one of the following: previous myocardial infarction, peripheral artery disease or complex aortic plaque.

### Echocardiographic data acquisition

All patients underwent a comprehensive imaging and Doppler echocardiographic examination using a commercially available system (Vivid 7 Dimension, GE Healthcare, Horten, Norway) according to our laboratory protocol. Examinations were performed by three trained transthoracic and transoesophageal echocardiographers, the images were stored on digital media for subsequent analysis using the commercially available software EchoPac Dimension PC version 108.1.4, GE Health Care, and were reviewed by two investigators.

**Figure 1:**
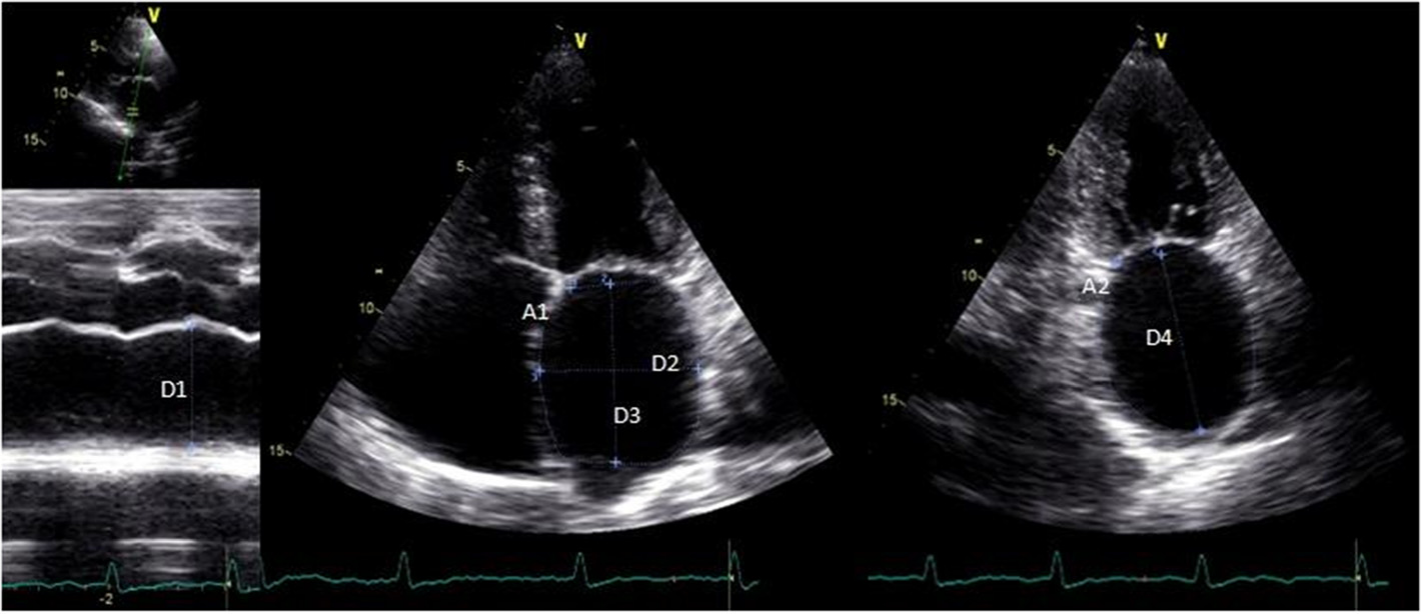
**Echocardiographic parameters used to calculate the left atrial volumes.** Legend: A1 - LA area in 4C view; A2 - LA area in 2C view; D1 - LA AP diameter; D2 - medial–lateral diameter; D3 - superior-inferior diameter in 4C view; D4 - superior-inferior diameter in 2C view.

M-mode and two-dimensional transthoracic images were acquired using a M4S probe (1.5–4.0 MHz), and were used to obtain the following LA measurements: LA AP diameter, LA area from 4-chamber view (4C), and LA volumes by the ellipsoid, single plane (1P) area-length and biplane (2P) area-length methods. These measurements were obtained at end-ventricular systole, from the frame immediately preceding mitral valve opening, as shown in Figure 1. LA AP diameter (D1) was measured by M-mode from the parasternal long-axis view. LA area was measured using planimetry in TTE apical four-chamber and two chamber view. LA ellipsoid volume was calculated using AP (D1), medial–lateral (D2) and superior–inferior (D3) LA diameters (Figure 1) and the formula: 4/3π*(D1/2) * (D2/2) * (D3/2). LA 1P area-length volume was obtained through the formula 8/3π*A1^2^/D3, where A1 represents the area and D3 the superior-inferior LA diameter measured from apical 4C view. LA 2P area-length volume was achieved with the formula 8/3 π*[(A1) * (A2)/L], where A2 represent the LA area 2-chamber (2C) view, and L the shortest superior-inferior diameter measured in 4C (D3) and 2C (D4) views (Figure 1). All these measurements were indexed to body surface area.

TEE images were acquired with a 6 T phased array multiplane transoesophageal probe (2.9–7.0 MHz). The LA and LAA were imaged in different tomographic planes to detect the presence of LAA thrombus and SEC. LA thrombus was diagnosed by the presence of an echo-dense mass in the left atrium or the LAA [28]. Spontaneous echocardiographic contrast was diagnosed by the presence of characteristic dynamic smoke-like swirling echoes in the LA or the LAA, distinct from background white noise caused by excessive gain [29], and was classified according to the classification (1 to 4+) proposed by Fatkin et al. [30]. Dense SEC was defined as grade 3+ or 4+. Left atrial appendage flow velocities were assessed with a pulsed Doppler sample placed 1 cm from the entry of the LAA into the body of the LA. Maximum emptying and filling velocities were estimated from an average of five well-defined emptying and filling waves. Patients with maximum emptying and filling velocity ≤20 cm/s were classified as having low flow velocities.

### Study endpoints

The study endpoints were the TEE surrogate markers of stroke: LAA thrombus, LAA low flow velocities and dense SEC. The composite endpoint of LA abnormality (LA ABN) was defined by the presence of at least one of the previous markers.

### Statistical analysis

Statistical analysis was performed using SPSS, version 17.0. Baseline characteristics were described with mean ± standard deviation for continuous data and counts and proportions for categorical data. The Kolmogorov-Smirnov test was used to test the normal distribution of continuous variables. The Chi-square test, Student’s *t*-test and non-parametric equivalent tests were used when appropriate. P values <0.05 (two-sided) were considered statistically significant.

A comparative univariate analysis of patients with and without LA ABN was performed to evaluate potential differences between the groups regarding demographic, clinical, echocardiographic and laboratorial parameters.

The discriminatory power of the indexed and non-indexed LA measurements (AP diameter, area 4C, ellipsoid volume, 1P area-length volume and 2P area-length volume) to identify TEE surrogate markers of stroke (LAA thrombus, LAA low flow velocities, dense SEC and LA ABN) was evaluated through the receiver operating characteristic (ROC) curve, which refers to the ability of a model to assign a higher probability to patients reaching an endpoint than those who did not reach it. Comparisons of areas under ROC curves (AUC) were performed between LA indexed measurements, using MedCalc for Windows version 9.2.0.1. A ROC curve subanalysis was also carried out for patients previously under oral anticoagulation regarding LA thrombus, LAA low flow velocities or at least one of these two parameters.

Finally, multivariate analysis (forward conditional method) was performed to identify independent predictors of TEE surrogate markers of stroke, among the different methods of LA size quantification and the parameters included in CHADS_2_ or CHA_2_DS_2_-VASc scores.

Inter- and intra-observer variability was assessed for the three echocardiographers using a sample composed of the first 50 patients included in the study: for dense SEC and LAA thrombus correlations were used; for LA measurements, we performed a Bland-Altman analysis using MedCalc for Windows version 9.2.0.1.

## Results

### Study population

Baseline characteristics of the 500 patients enrolled in this study are summarized in Table 1. There was a lower prevalence of female gender (34.2%). The average CHADS_2_ and CHA_2_DS_2_-VASc scores were 1.9 ± 1.2 and 3.2 ± 1.7, respectively. Forty point six per cent of patients were previously medicated with oral anticoagulants and 33.8% with antiplatelet agents; in 33.8% of patients, one to three injections of enoxaparin were performed prior to TEE. The duration of the current atrial fibrillation episode was estimated to be longer than one year in 26.8% of patients (Table 1). AF was persistent in 73.2% of patients (n = 366) and long-standing persistent in 26.8% of patients (n = 134). TEE examinations identified 55 patients (11.1%) with LAA thrombus, 66 (13.2%) with low LAA flow velocities and 120 (24%) with dense SEC. LA ABN was present in 29.6% of patients (n = 148).

**Table 1:** Study population baseline characteristics

**Baseline characteristics (n = 500)**			
**Demographics**		**Medication previous to TEE**	
Age (years)	69 ± 10	Oral Anticoagulation	40.6% (203)
Age ≥ 75 years	31% (155)	Antiplatelet agents	46.4% (232)
Age 65–74 years	39.8% (199)	1-3 doses of enoxaparine	33.8% (169)
Body Mass Index (Kg/m^2^)	26.6 ± 8.8	**Echocardiographic characterization**	
Female gender	34.2% (171)	LA AP diameter (cm)	4.7 ± 0.7
**Clinical data**		LA area (cm^2^)	27.6 ± 7.1
Hypertension	81.8% (409)	LA ellipsoid volume (cm^3^)	74.1 ± 29.0
Diabetes	23.6% (118)	LA 1P area-length volume (cm^3^)	113.8 ± 47.7
Previous Stroke/TIA	14.8% (74)	LA 2P area-length volume (cm^3^)	104.1 ± 38.1
Congestive heart failure	54.8% (274)	LV ejection fraction ≥55%	73.6% (368)
Vascular disease^a^	52% (260)	LAA thrombus	11.1% (55)
CHADS_2_	1.9 ± 1.2	LAA low flow velocities	13.2% (66)
CHA_2_DS_2_-VASc	3.2 ± 1.7	Dense SEC	24% (120)
Pacemaker or ICD	19.6% (98)	LA ABN	29.6% (148)
**Estimated current AF episode duration**		**Laboratorial evaluation**	
≤ 48 hours	7% (35)	Haemoglobin (g/dL)	13.2 ± 1.8
< 1 week	19.6% (98)	Platelets (10^3^/μL)	219.7 ± 85.5
< 1 month	36% (180)	INR	1.5 ± 0.8
> 6 months	35.8% (179)	INR ≥ 2.0	20.8% (104)
> 1 year	26.8% (134)	GFR (mL/min/1.73 m^2^)	62.4 ± 33.0

Legend: GFR – glomerular filtration rate; ICD - implantable cardioverter defibrillator; INR – international normalized ratio; LV – left ventricle; TIA – transient ischaemic attack.

^a^Vascular disease is defined as having at least one of the following: myocardial infarctions, peripheral artery disease, and complex aortic plaque.

The sub-analysis of demographic, clinical, echocardiographic and laboratorial characteristics of the study population according to the presence or absence of LA ABN is presented in Table 2. Patients with LA ABN had higher thromboembolic risk (CHADS_2_: 2.1 ± 1.1 vs. 1.8 ± 1.2, p = 0.001; CHA_2_DS_2_-VASc: 3.6 ± 1.6 vs. 3.0 ± 1.7, p < 0.001), were older (70 ± 8 vs. 68 ± 11, p = 0.02), had higher prevalence of congestive heart failure (65.7% vs. 50.4%, p = 0.002) and vascular disease (62.8% vs. 47.4%, p = 0.003), and less frequently a left ventricle ejection fraction ≥55% (58.8% vs. 79.8%, p < 0.001). They presented higher values for all LA measurements performed and had longer AF duration (Table 2). Although patients with LA ABN were more often anticoagulated (50% vs. 36.6%, p = 0.01), there was no difference in the prevalence of effective anticoagulation (20.9% vs. 20.7%, p = ns) or in the average INR (1.5 ± 0.7 vs. 1.5 ± 0.8, p = ns) at the time TEE was performed.

**Table 2:** Sub-analysis of baseline characteristics according to the presence of left atrial abnormality

**Parameter**	**Without LA ABN**	**With LA ABN**	**p**
**Demographics**			
Age (years)	68 ± 11	70 ± 8	0.02
Body Mass Index (Kg/m^2^)	26.8 ± 9	26 ± 8.1	0.4
Female gender	33% (116)	37.2% (55)	0.4
**Clinical data**			
Hypertension	80.8% (284)	84.6% (125)	0.4
Diabetes	21.9% (77)	28% (41)	0.1
Previous Stroke/TIA	13.2% (46)	18.9% (28)	0.1
Congestive heart failure	50.4% (177)	65.7% (97)	0.002
Vascular disease^a^	47.4% (167)	62.8% (93)	0.003
CHADS_2_ score	1.8 ± 1.2	2.1 ± 1.1	0.001
CHA_2_DS_2_-VASc score	3 ± 1.7	3.6 ± 1.6	<0.001
Pacemaker or ICD	18.8% (66)	21.7% (32)	0.7
**Estimated current AF episode duration**			
≤ 48 hours	8.5% (30)	3.6% (5)	0.06
< 1 week	24% (84)	9.5% (14)	<0.001
< 1 month	40% (141)	26.3% (39)	0.001
> 6 months	31% (109)	47.3% (70)	0.001
> 1 year	22.2% (78)	37.8% (51)	0.001
**Echocardiographic characterization**			
LA AP diameter, (cm)	4.6 ± 0.7	4.8 ± 0.8	0.004
LA area (cm^2^)	26.4 ± 6.8	30.3 ± 7.1	<0.001
LA ellipsoid volume (cm^3^)	70.5 ± 28.2	82.1 ± 29.4	0.001
LA 1P area-length volume (cm^3^)	106.6 ± 45.9	130 ± 47.7	<0.001
LA 2P area-length volume (cm^3^)	97.6 ± 37.6	117 ± 35.9	<0.001
Indexed LA AP diameter, (cm/m^2^)	2.4 ± 0.5	2.7 ± 0.4	<0.001
Indexed LA area (cm^2^/m^2^)	14.2 ± 4.1	16.9 ± 4.1	<0.001
Indexed LA ellipsoid volume (cm^3^/m^2^)	37.6 ± 16.3	45.3 ± 16.3	<0.001
Indexed LA 1P area-length volume (cm^3^/m^2^)	56.9 ± 24.7	72.3 ± 26.9	<0.001
Indexed LA 2P area-length volume (cm^3^/m^2^)	51.9 ± 20.6	64.8 ± 20.9	<0.001
LV ejection fraction ≥55%	79.8% (281)	58.8% (87)	<0.001
**Medication previous to TEE**			
Oral Antiocagulation	36.6% (129)	50% (74)	0.01
Antiplatelet agents	48.8% (172)	40.5% (60)	0.1
1-3 doses of enoxaparine	36.1% (127)	28.3% (42)	0.1
**Laboratorial evaluation**			
Haemoglobin (g/dL)	13.3 ± 1.9	13.1 ± 1.6	0.3
Platelets (10^3^/μL)	220.6 ± 91.4	217.5 ± 69.4	0.7
INR	1.5 ± 0.8	1.5 ± 0.7	0.8
INR ≥ 2.0	20.7% (73)	20.9% (31)	0.96
GFR (mL/min/1.73 m^2^)	61.9 ± 33.1	63.8 ± 32.8	0.6

Legend: GFR – glomerular filtration rate; ICD - implantable cardioverter defibrillator; INR – international normalized ratio; LV – left ventricle; TIA – transient ischaemic attack.

^a^Vascular disease is defined as having at least one of the following: myocardial infarctions, peripheral artery disease, and complex aortic plaque.

### LA measurements for identifying surrogate markers of stroke on TEE

**Figure 2:**
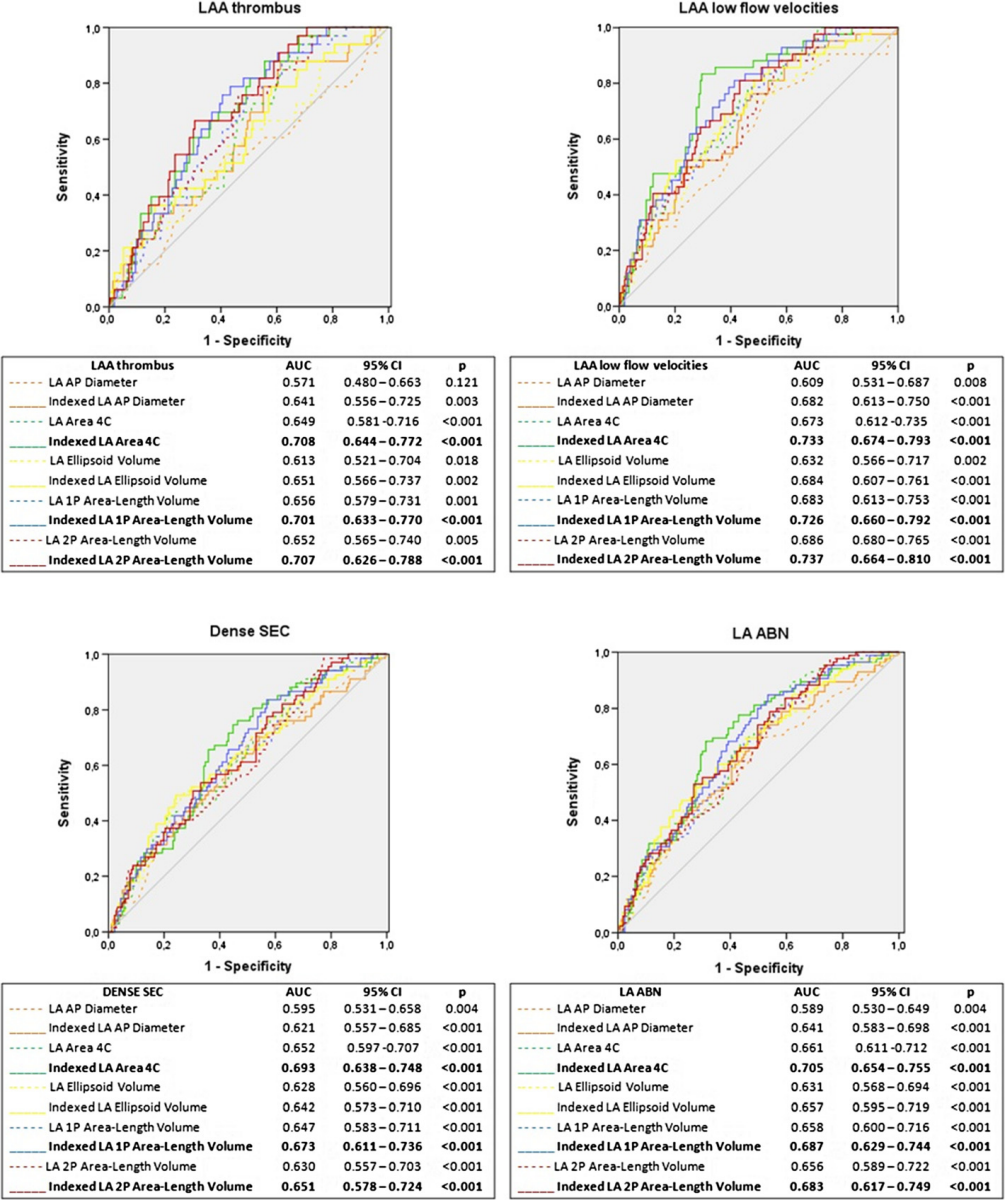
**Receiver operating characteristic-curve analysis of indexed and non-indexed left atrial measurements for identifying TEE endpoints.** Legend: AP – anteroposterior; AUC – area under the curve; CI – confidence interval; LA – left atrial; LAA – left atrial appendage; LA ABN – left atrial abnormality; p – significance level; SEC – spontaneous echocardiographic contrast; 4C – 4-chamber; 1P – single-plane; 2P – biplane.

**Figure 3:**
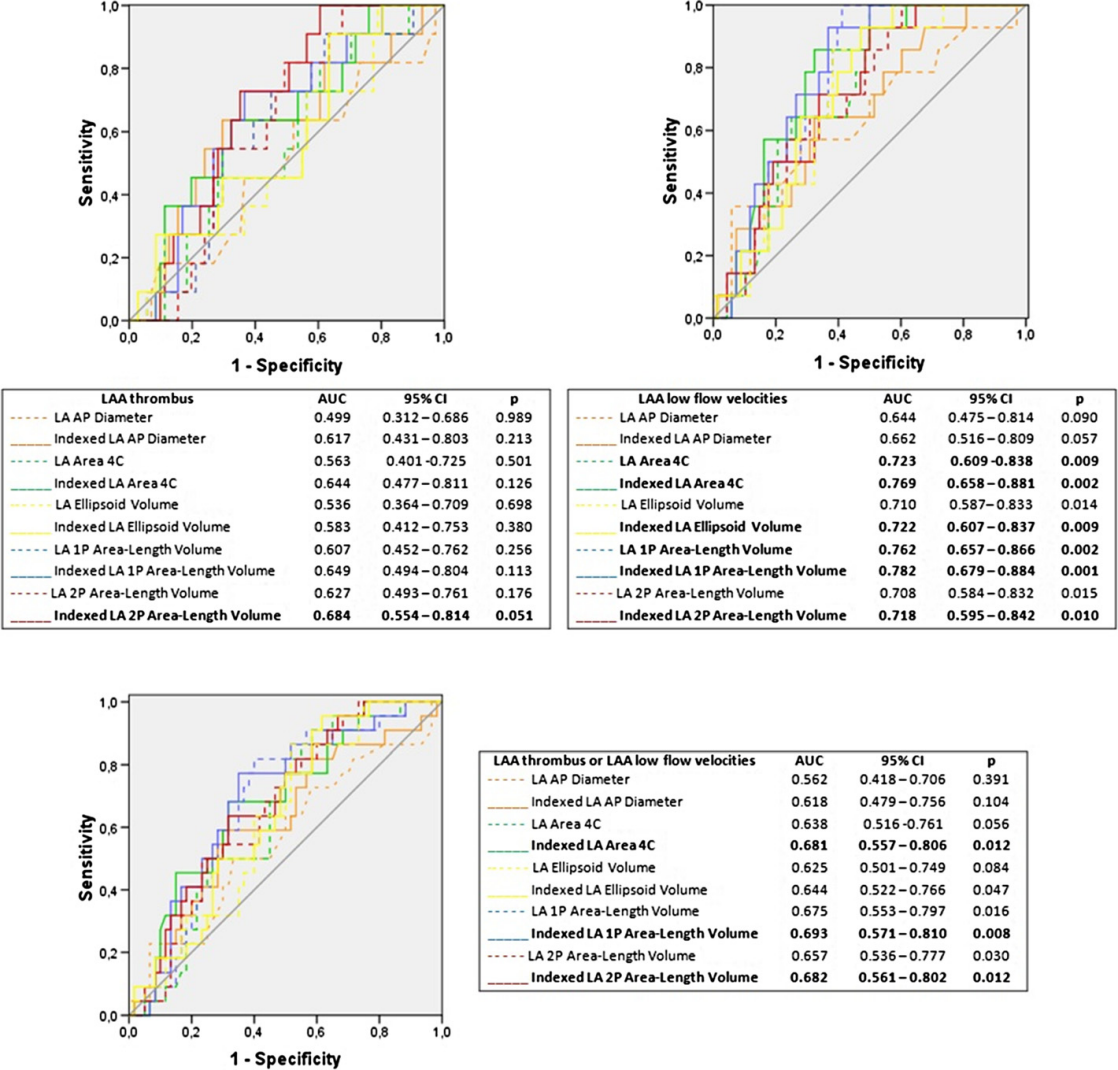
**Receiver operating characteristic-curve sub-analysis of left atrial measurements for identifying LAA thrombus, LAA low flow velocities or at least one of one of these parameters, in patients under previous oral anticoagulation.** Legend: AP – anteroposterior; AUC – area under the curve; CI – confidence interval; LA – left atrial; LAA – left atrial appendage; p – significance level; 4C – 4-chamber; 1P – single-plane; 2P – biplane.

Figure 2 presents the results of ROC curve analysis of the different indexed and non-indexed LA measurements for identifying LAA thrombus, LAA low flow velocities, dense SEC and LA ABN.Except for LA AP diameter in identifying LA thrombus, all other LA measurements showed a good discriminatory ability in the prediction of the study endpoints (Figure 2). Indexing to body surface area increased the accuracy of every LA measurement to recognize each TEE surrogate marker of stroke (Figure 2).Taking into account the possible influence of oral anticoagulation on dense SEC, a sub-analysis of the discriminatory ability of LA measurements for predicting the other surrogate markers of stroke (LA thrombus, LAA low flow velocities or at least one of these two parameters), was carried out for patients previously under oral anticoagulation (Figure 3). In this subset of 165 patients, LAA thrombus was found in 21 patients, LAA low flow velocities in 23 patients and at least one of these parameters in 40 patients. There was a good discriminatory ability of the LA measurements for identifying LAA low flow velocities in this sample, though the results were not as consistent for identifying LAA thrombus (Figure 3).

### The most suitable TTE measurements to identify TEE surrogate markers of stroke

The highest AUC for the prediction of study endpoints was achieved with indexed LA area 4C for LAA thrombus (AUC 0.788, CI_95%_ 0.644 – 0.772, p < 0.001), dense SEC (AUC 0.693, CI_95%_ 0.638 – 0.748, p < 0.001) and LA ABN (AUC 0.705, CI_95%_ 0.654 – 0.755, p < 0.001). Regarding LAA low flow velocities, the highest AUC was obtained with indexed 2P area-length method (AUC 0.737, CI_95%_ 0.664 – 0.810, p < 0.001) – Figure 2.

Generally, indexed LA area 4C and indexed 1P and 2P area-length methods showed a moderately high discriminatory power in the prediction of the study endpoints and stood out from all the other measurements. ROC curve comparison for these three measurements did not reveal a significant difference between them (Table 3).

**Table 3:** Cross Tabulation of ROC AUC between the different indexed methods

**Indexed LA AP diameter**	**Indexed LA Area 4C**	**Indexed LA elipsoid volume**	**Indexed LA 1P area-length volume**	**Indexed LA 2P area-length volume**
**Indexed LA AP diameter**	LAAT p = 0.08	LAAT p = 0.910	LAAT p = 0.122	LAAT p = 0.082
	DSEC p = 0.1	DSEC p = 0.231	DSEC p = 0.248	DSEC p = 0.389
	**LFV p = 0.024**	LFV p = 0.358	LFV p = 0.127	LFV p = 0.245
	**LA ABN p = 0.046**	LA ABN p = 0.187	LA ABN p = 0.154	LA ABN p = 0.249
	**Indexed LA Area 4C**	**LAAT p = 0.037**	LAAT p = 0.888	LAAT p = 0.795
		DSEC p = 0.451	DSEC p = 0.466	DSEC p = 0.358
		LFV p = 0.064	LFV p = 0.061	LFV p = 0.069
		LA ABN p = 0.212	LA ABN p = 0.186	LA ABN p = 0.242
		**Indexed LA elipsoide volume**	**LAAT p = 0.010**	**LAAT p = 0.033**
			DSEC p = 0.722	DSEC p = 0.997
			LFV p = 0.314	LFV p = 0.472
			LA ABN p = 0.565	LA ABN p = 0.805
			**Indexed LA 1P area-length volume**	LAAT p = 0.766
				DSEC p = 0.608
				LFV p = 0.519
				LA ABN p = 0.633
				**Indexed LA 2P area-length volume**

Legend: AP – anteroposterior; DSEC – dense spontaneous echocardiographic contrast; LA – left atrial; LA ABN – left atrial abnormality; LAAT – left atrial appendage thrombus; LFV - left atrial appendage low flow velocities; p – significance level; 4C – 4-chamber; 1P – single-plane; 2P – biplane.

On multivariate analysis considering the different methods of LA size quantification and the parameters included in CHADS_2_ score, indexed LA area 4C was an independent predictor of LAA low flow velocities, dense SEC and LA ABN, while indexed 2P area-length volume was an independent predictor of LA thrombus (Table 4). The same analysis with the parameters of CHA_2_DS_2_-VASc score reached similar results, with exception for dense SEC (Table 4).

**Table 4:** **Multivariate analysis including LA measurements and CHADS**_**2**_**/CHA**_**2**_**DS**_**2**_**-VASc parameters for prediction of TEE surrogate markers of stroke**

**TEE markers of stroke**	**Independent predictors**	**OR**	**CI**_**95%**_	**p**
**LA measurements + CHADS**_**2 **_**parameters**				
**LAA thrombus**	• Indexed LA 2P Area-Length Volume	1.026	1.009 - 1.043	0.003
	• Previous stroke/TIA	4.080	1.647 – 10.102	0.002
**LAA LFV**	• Indexed LA Area 4C	1.174	1.083 – 1.272	<0.001
**Dense SEC**	• Indexed LA Area 4C	1.090	1.018 – 1.167	0.01
**LA ABN**	• Indexed LA Area 4C	1.114	1.037 – 1.196	0.003
	• Congestive heart failure	2.022	1.109 – 3.690	0.02
**LA measurements + CHA**_**2**_**DS**_**2**_**-VASc parameters**				
**LAA thrombus**	• Indexed LA 2P Area-Length Volume	1.028	1.010 – 1.046	0.002
	• Previous stroke/TIA	4.071	1.568 – 10.569	0.004
**LAA LFV**	• Indexed LA Area 4C	1.186	1.089 – 1.291	<0.001
**Dense SEC**	• Previous stroke/TIA	2.675	1.158 – 6.180	0.02
	• Congestive heart failure	2.308	1.180 – 4.514	0.02
**LA ABN**	• Indexed LA Area 4C	1.106	1.029 – 1.190	0.006
	• Congestive heart failure	2.097	1.126 – 3.907	0.02

Legend: CI - confidence interval; LA – left atrial; LAA – left atrial appendage; LA ABN – left atrial abnormality; LFV – low flow velocities; OR – odds ratio; p – significance level; SEC – spontaneous echocardiographic contrast; TEE – transesophageal echocardiography; TIA – transient ischemic attack; vel – velocities; 4C – 4-chamber; 2P – biplane.

On multivariate analysis including the LA volumes and the CHADS_2_ score parameters, indexed 2P area-length volume was an independent predictor of LA thrombus, dense SEC and LA ABN; Indexed 1P area-length volume was independent predictor of LAA low flow velocities (Table 5). The same analysis considering CHA_2_DS_2_-VASc score parameters reached similar results, with exception for dense SEC (Table 5).

**Table 5:** **Multivariate analysis including LA volume measurements and CHADS**_**2**_**/CHA**_**2**_**DS**_**2**_**-VASc parameters for prediction of TEE surrogate markers of stroke**

**TEE markers of stroke**	**Independent predictors**	**OR**	**CI**_**95%**_	**p**
**LA Volumes + CHADS**_**2 **_**parameters**				
**LAA thrombus**	• Indexed LA 2P Area-Length Volume	1.026	1.009 – 1.043	0.003
	• Previous stroke/TIA	4.080	1.647 – 10.102	0.002
**LAA LFV**	• Indexed LA 1P Area-Length Volume	1.022	1.010 – 1.034	<0.001
**Dense SEC**	• Indexed LA 2P Area-Length Volume	1.013	0.999-1.028	0.07
	• Congestive heart failure	1.968	1.024 – 3.783	0.04
	• Previous stroke/TIA	2.458	1.093 – 5.530	0.03
**LA ABN**	• Indexed LA 2P Area-Length Volume	1.021	1.006 – 1.035	0.005
	• Congestive heart failure	2.065	1.134 – 3.758	0.02
**LA Volumes + CHA**_**2**_**DS**_**2**_**-VASc parameters**				
**LAA thrombus**	• Indexed LA 2P Area-Length Volume	1.028	1.010 – 1.046	0.002
	• Previous stroke/TIA	4.071	1.568 – 10.569	0.004
**LAA LFV**	• Indexed LA 1P Area-Length Volume	1.023	1.180 – 1.037	<0.001
**Dense SEC**	• Previous stroke/TIA	2.675	1.158 – 6.180	0.02
	• Congestive heart failure	2.308	1.180 – 4.514	0.02
**LA ABN**	• Indexed LA 2P Area-Length Volume	1.019	1.005 – 1.034	0.009
	• Congestive heart failure	2.133	1.147 – 3.966	0.02

Legend: CI - confidence interval; LA – left atrial; LAA – left atrial appendage; LA ABN – left atrial abnormality; LFV – low flow velocities; OR – odds ratio; p – significance level; SEC – spontaneous echocardiographic contrast; TEE – transesophageal echocardiography; TIA – transient ischemic attack; vel – velocities; 1P – single-plane; 2P – biplane.

A multivariate analysis taking into account LA areas and CHADS_2_ or CHA_2_DS_2_-VASc score parameters revealed indexed LA area 4C as an independent predictor of all TEE endpoints (Table 6).

**Table 6:** **Multivariate analysis including LA area measurements and CHADS**_**2**_**/CHA**_**2**_**DS**_**2**_**-VASc parameters for prediction of TEE surrogate markers of stroke**

**TEE markers of stroke**	**Independent predictors**	**OR**	**CI**_**95%**_	**p**
**LA Areas + CHADS**_**2 **_**parameters**				
**LAA thrombus**	• Indexed LA Area 4C	1.119	1.047 - 1.196	0.001
	• Previous stroke/TIA	3.977	1.987 – 7.959	<0.001
**LAA LFV**	• Indexed LA Area 4C	1.141	1.075 – 1.211	<0.001
**Dense SEC**	• Indexed LA Area 4C	1.136	1.078 – 1.198	<0.001
**LA ABN**	• Indexed LA Area 4C	1.142	1.083 – 1.204	<0.001
	• Congestive heart failure	1.592	1.019 – 2.487	0.04
**LA Areas + CHA**_**2**_**DS**_**2**_**-VASc parameters**				
**LAA thrombus**	• Indexed LA Area 4C	1.133	1.058 – 1.213	<0.001
	• Previous stroke/TIA	3.932	1.917 – 8.069	<0.001
**LAA LFV**	• Indexed LA Area 4C	1.139	1.071– 1.212	<0.001
**Dense SEC**	• Indexed LA Area 4C	1.125	1.065 – 1.189	<0.001
	• Previous Vascular disease^a^	1.930	1.180 – 3.154	0.009
**LA ABN**	• Indexed LA Area 4C	1.147	1.086 – 1.210	<0.001
	• Previous Vascular disease^a^	1.581	1.010 – 2.474	0.05

Legend: CI - confidence interval; LA – left atrial; LAA – left atrial appendage; LA ABN – left atrial abnormality; LFV – low flow velocities; OR – odds ratio; p – significance level; SEC – spontaneous echocardiographic contrast; TEE – transesophageal echocardiography; TIA – transient ischemic attack; vel – velocities; 4C – 4-chamber.

^a^Vascular disease is defined as having at least one of the following: myocardial infarctions, peripheral artery disease, and complex aortic plaque.

Inter- and intra-observer variability for the different LA size quantification methods, dense SEC and LAA thrombus is presented in Table 7.

**Table 7:** Inter- and intra-observer variability for the different LA quantification methods, dense spontaneous spontaneous echocardiographic contrast and left atrial appendage thrombus

**Inter-observer variability**	**A vs B**	**A vs C**	**B vs C**
LA AP Diameter (mean ± SD,%)	1.05 ± 0.19	1.04 ± 0.2	1.04 ± 0.18
LA Area 4C (mean ± SD,%)	1.03 ± 0.23	1.04 ± 0.24	1.02 ± 0.21
LA Ellipsoid Volume (mean ± SD,%)	1.12 ± 0.36	1.1 ± 0.36	1.08 ± 0.33
LA 1P Area-Lenght Volume (mean ± SD,%)	1.09 ± 0.44	1.11 ± 0.47	1.06 ± 0.41
LA 2P Area-Lenght Volume (mean ± SD,%)	1.05 ± 0.28	1.07 ± 0.28	1.03 ± 0.27
Dense SEC	R = 0.797, p < 0.001	R = 0.849, p < 0.001	R = 0.841, p < 0.001
LAA Thrombus	R = 0.857, p < 0.001	R =1, p < 0.001	R = 0.857, p < 0.001
**Intra-observer variability**	**A**	**B**	**C**
LA AP Diameter (mean ± SD,%)	1 ± 0.08	1 ± 0.08	1 ± 0.07
LA Area 4C (mean ± SD,%)	1.01 ± 0.11	1.01 ± 0.09	1.01 ± 0.1
LA Ellipsoid Volume (mean ± SD,%)	1.01 ± 0.1	01.01 ± 0.1	0.98 ± 0.11
LA 1P Area-Lenght Volume (mean ± SD,%)	1.03 ± 0.24	1.02 ± 0.2	1.03 ± 0.22
LA 2P Area-Lenght Volume (mean ± SD,%)	1.01 ± 0.15	1.01 ± 0.15	1.02 ± 0.14
Dense SEC	R =0.901, p < 0.001	R =1, p < 0.001	R = 1, p < 0.001
LAA Thrombus	R = 1, p < 0.001	R = 1, p < 0.001	R =1, p < 0.001

Legend: LA – left atrial; LAA – left atrial appendage; SEC – spontaneous echocardiographic contrast; 4C – 4-chamber; 1P – single-plane; 2P – biplane.

A, B and C represent the three different echocardiographers.

For LA measurements are presented the results of Bland-Altman analysis, expressed as mean ± SD of the ratio of the measurements in comparison. For dense spontaneous spontaneous echocardiographic contrast and left atrial appendage thrombus are presented the variability is presented as a correlation.

## Discussion

Our results suggest that LA dilation is associated with an increase in the prevalence of TEE markers of increased thromboembolic risk in patients with AF, independently from recognized clinical risk factors. A stronger association was found for measurements indexed to body surface area, with the best discriminative ability obtained with indexed LA area 4C, indexed LA 1P area-length volume and indexed LA 2P area-length volume.

This is the first study to date evaluating and comparing the discriminative power of different methods for LA size measurement in the prediction of markers of left atrial stasis: LAA thrombus, dense SEC, LAA low flow velocities and LA ABN.

### Predictors of left atrial stasis

The presence of thrombus in the LAA, dense SEC, LAA low flow velocities and LA ABN are strongly associated with thromboembolism and adverse outcomes in patients with AF [22-24]. Accordingly, TEE has been recommended to evaluate the risk of thromboembolism previous to procedures such as cardioversion or catheter ablation [31,32].

In this study population, the presence of LA ABN was associated with longer AF duration and higher thromboembolic risk assessed by CHADS_2_ and CHA_2_DS_2_-VASc scores, due to a higher prevalence of recognized thromboembolic risk factors such as older age, heart failure, vascular disease and systolic dysfunction (Table 2). LA ABN was also associated with a larger left atrium, independently of the used method (Table 2). On multivariate analysis, indexed LA area 4C and indexed 2P area-length volume were predictors of LA ABN (Tables 4, 5 and 6). It is now established that LA enlargement is not considered part of the normal aging process, but related to underlying pathological processes such as systolic dysfunction, heart failure or vascular disease.

### Left atrial size – an underexplored marker of prothrombotic risk?

For all evaluated methods, a larger LA was associated to LA ABN (Table 2). Also, most non-indexed and all indexed measurements showed moderate accuracy in the prediction of all thromboembolic risk markers assessed by TEE (Figure 2). Similar results were also found for identifying LAA low flow velocities or at least LAA low flow velocities or LAA thrombus in the subset of patients previously under oral anticoagulation. For predicting LAA thrombus in this subgroup, the results were not so consistent among the different LA measurements, however they may have been influenced by the small number of patients (n = 165) and the limited number of surrogate markers of stroke (LAA Thrombus: n = 21, low LAA flow velocities: n = 23, both n = 40) included in this sample.

On multivariate analysis, LA size was predictor of LA ABN, LAA thrombus, LAA low flow velocities and dense SEC independently from most thromboembolic risk factors used in CHADS_2_ and CHA_2_DS_2_-VASc scores (Tables 4, 5 and 6). These results suggest that LA size may be used to predict stroke in patients with non-valvular AF and, eventually, improve thromboembolic risk stratification of these patients. In one study of 334 patients with AF undergoing transesophageal echocardiography, indexed LA volume was a significant predictor of LAA thrombus (OR 1.02, p = 0.02) and added incremental predictive value to left ventricle ejection fraction [33]. Another study demonstrated that LA area 4C associated to left ventricular ejection fraction could be used to identify LAA thrombus, LAA low flow velocities, dense SEC or LA ABN, and further refine the ability of CHADS_2_ and CHA_2_DS_2_-VASc scores to identify these TEE surrogate markers of stroke [34]. However, the discriminative capability of an isolated LA measurement to identify TEE surrogate markers of stroke has never been evaluated.

Due to the wide variety of LA size quantification methods, it seems important to identify the most suitable and accurate method for thromboembolic risk assessment. When assessing LA size it is crucial to index results to body surface area, as confirmed by the observed improvement in AUC with the different indexed methods in our sample.

The indexed measurements of LA area 4C, LA 1P and 2P area-length volumes stood out among all LA measurements for their moderate to high discriminatory power in the prediction of LAA thrombus, LAA low flow velocities, dense SEC and LA ABN (Figure 2), without significant differences between them (Table 3). Indexed LA area 4C was an independent predictor of all TEE endpoints (Table 6). Importantly, for dense SEC, LAA low flow velocities and LA ABN, this predictive ability was independent of other LA measurements (Table 4). For LAA thrombus, indexed 2P area-length volume was a predictor of TEE surrogate markers of stroke, irrespectively of the other LA measurements (Table 4). When considered separately from LA area 4C, the indexed LA area-length volumes also showed a good predictive ability for TEE surrogate markers of stroke (Table 5).

LA enlargement is often asymmetrical, as enlargement in AP axis may be limited by the thoracic cavity, and it may also occur in medial-lateral or superior-inferior axes [11,14]. Thus, LA AP diameter has been considered inaccurate, while LA areas or volumes assessed by TTE rely on fewer geometric assumptions. The 2P volume, using either the area-length or the discs formula, is the method currently recommended for LA size quantification [19]. In our study, this indexed method has also proved to be one of the most suitable to indicate the presence of TEE surrogate markers of stroke, yet single-plane area-length and area 4C methods showed a similar ability for this purpose.

Russo C and colleagues [13] evaluated single-plane and biplane methods for the assessment of LA volume against three-dimensional echocardiography in 527 participants of a community-based Cohort. They found strong correlations between single- and biplane LA volume measurements (r = 0.95, p < 0.01), and single- (r = 0.93, p < 0.01) and biplane (r = 0.93, p < 0.01) area-length with three-dimensional volumes, although single-plane method had a suboptimal agreement for categorical classification. They suggested that single-plane method could simplify and expedite LA volume evaluation, but specific cut-off points should be developed for this method.

Similar results were also found for LA area 4C and single-plane area-length methods in the study of Badano LP and colleagues [15], who found an excellent correlation between LA area 4C (r = 0.94, p < 0.0001), single-plane volume (r = 0.98, p < 0.0001) and biplane volume (r = 0.99, p < 0.0001) with volumes determined by three-dimensional echocardiography. They verified that the various methods of LA size quantification were not comparable, with a better agreement for single- and biplane volume methods, while LA area misled LA dilatation severity. They concluded that LA area inaccuracy was due mainly to inappropriate cut-off values than to the parameter itself. Furthermore, they suggested that the small additional accuracy obtained by using the biplane instead of the single-plane area–length method could allow the use of the single-plane method for routine clinical use [15]. Interestingly, reference ranges are also not available for indexed LA area 4C method in the last joint recommendations for chamber quantification [19].

In our study we found no significant differences when comparing indexed LA area 4C, LA 2P and 1P area-length volume for the discrimination of TEE markers of thromboembolic risk. This may be due to fact that differences between methods may be small and our sample may lack the necessary statistical power to demonstrate them. Further studies, sufficiently powered to assess this issue may be needed. However, at present moment there is no evidence suggesting that one should be preferred over the others for this specific aim.

### Limitations

There are several limitations in this study that must be highlighted. First, this was a single-centre study, whose sample was not powered for enabling comparisons between some of the different methods used for assessing LA size.

Second, at the time of TEE, 40.6% of the study population was under oral anticoagulation, which may have had an impact in the prevalence of LA stasis markers. However, we believe this study cohort is representative of the general population of patients with AF and, as such, we decided not to exclude patients under oral anticoagulation. Moreover, some of these patients were not under therapeutic INR values and it is known that thrombi may arise even under therapeutic INR.

## Conclusion

LA enlargement, determined by indexed LA AP diameter, and indexed and non-indexed LA area 4C and volumes, is suitable to identify LAA thrombus, LAA low flow velocities, dense SEC and LA ABN in patients with non-valvular AF. The indexed measurements of LA area 4C, single- and biplane area-length volumes are the most accurate methods for this purpose, and may be used to stratify thromboembolic risk in these patients.

## Abbreviations

AF: Atrial fibrillation; AP: Anteroposterior, for diameter measured by M-mode; AUC: Area under receiver operating characteristic curve; LA: Left atrial; LAA: Left atrial appendage; LA ABN: Left atrial abnormality; ROC curve: Receiver operating characteristic curve; SEC: Spontaneous echocardiographic contrast; TEE: Two-dimensional transesophageal echocardiography; TTE: Two-dimensional transthoracic echocardiography; 2C: Two-chamber view; 4C: Four-chamber view; 1P: Single-plane; 2P: Biplane.

## Competing interests

The authors declare that they have no competing interests.

## Authors’ contributions

AF: conception and design, acquisition, analysis and interpretation of data, draft of the manuscript; RP: design, acquisition, analysis and interpretation of data, critical review of the manuscript; SB: critical review of the manuscript; LP: acquisition, analysis and interpretation of data; JT: acquisition of echocardiography images; AB: acquisition of echocardiography images; MC: gave final approval of the version to be published; LG: gave final approval of the version to be published. All authors have read and approved the final version of the manuscript and provided insight for its elaboration.

## References

[B1] Benjamin EJ D’Agostino RB Belanger AJ Wolf PA Levy D Left atrial size and the risk of stroke and death. The Framingham Heart Study Circulation 1995 92 835 841 7641364 10.1161/01.cir.92.4.835

[B2] Tsang T Barnes M Bailey K Leibson CL Montgomery SC Takemoto Y Diamond PM Marra MA Gersh BJ Wiebers DO Petty GW Seward JB Left atrial volume: important risk marker of incident atrial fibrillation in 1655 older men and women Mayo Clin Proc 2001 76 467 475 11357793 10.4065/76.5.467

[B3] Barnes ME Miyasaka Y Seward JB Gersh BJ Rosales AG Bailey KR Petty GW Wiebers DO Tsang TS Left atrial volume in the prediction of first ischemic stroke in an elderly cohort without atrial fibrillation Mayo Clin Proc 2004 79 1008 1014 15301328 10.4065/79.8.1008

[B4] Tsang TS Gersh BJ Appleton CP Tajik AJ Barnes ME Bailey KR Oh JK Leibson C Montgomery SC Seward JB Left ventricular diastolic dysfunction as a predictor of the first diagnosed nonvalvular atrial fibrillation in 840 elderly men and women J Am Coll Cardiol 2002 40 1636 1644 12427417 10.1016/s0735-1097(02)02373-2

[B5] Tsang TS Barnes ME Gersh BJ Bailey KR Seward JB Risks for atrial fibrillation and congestive heart failure in patients ≥ 65 years of age with abnormal left ventricular diastolic relaxation Am J Cardiol 2004 93 54 58 14697466 10.1016/j.amjcard.2003.09.012

[B6] Aronow WS Gutstein H Hsieh FY Risk factors for thromboembolic stroke in elderly patients with chronic atrial fibrillation Am J Cardiol 1989 63 366 367 2783633 10.1016/0002-9149(89)90349-4

[B7] Aronow WS Ahn C Kronzon I Gutstein H Risk factors for new thromboembolic stroke in patients > or = 62 years of age with chronic atrial fibrillation Am J Cardiol 1998 82 119 121 9671020 10.1016/s0002-9149(98)00247-1

[B8] Osranek M Bursi F Bailey KR Grossardt BR Brown RD Kopecky SL Tsang TS Seward JB Left atrial volume predicts cardiovascular events in patients originally diagnosed with lone atrial fibrillation: three-decade follow-up Eur Heart J 2005 26 2556 2561 16141257 10.1093/eurheartj/ehi483

[B9] Caplan LR D'Cruz I Hier DB Reddy H Shah S Atrial size, atrial fibrillation and stroke Ann Neurol 1986 19 158 161 3963758 10.1002/ana.410190208

[B10] Vaziri SM Larson MG Benjamin EJ Levy D Echocardiographic predictors of nonrheumatic atrial fibrillation. The Framingham Heart Study Circulation 1994 89 724 730 8313561 10.1161/01.cir.89.2.724

[B11] Leung DY Boyd A Arnold H Chi C Thomas L Echocardiographic evaluation of left atrial size and function: current understanding, pathophysiologic correlates, and prognostic implications Am Heart J 2008 156 1056 1064 19032999 10.1016/j.ahj.2008.07.021

[B12] Tsang TS Abhayaratna WP Barnes ME Miyasaka Y Gersh BJ Bailey KR Cha SS Seward JB Prediction of cardiovascular outcomes with left atrial size. Is volume superior to area or diameter? J Am Coll Cardiol 2006 47 1018 1023 16516087 10.1016/j.jacc.2005.08.077

[B13] Russo C Hahn RT Jin Z Homma S Sacco RL Di Tullio MR Comparison of echocardiographic single- vs biplane method in the assessment of left atrial volume and validation by real time three-dimensional echocardiography J Am Soc Echocardiogr 2010 23 9 954 960 20650605 10.1016/j.echo.2010.06.010 PMC2929292

[B14] Abhayaratna WP Seward JB Appleton CP Douglas PS Oh JK Tajik AJ Tsang TSM Left atrial size: physiologic determinants and clinical applications J Am Coll Cardiol 2006 47 2357 2363 16781359 10.1016/j.jacc.2006.02.048

[B15] Badano LP Pezzutto N Marinigh R Cinello M Nucifora G Pavoni D Gianfagna P Fioretti PM How many patients would be misclassified using M-mode and two-dimensional estimates of left atrial size instead of left atrial volume? A three-dimensional echocardiographic study J Cardiovasc Med 9 476 484 10.2459/JCM.0b013e3282f194f0 18403999

[B16] Maddukuri PV Vieira ML DeCastro S Maron MS Kuvin JT Patel AR Pandian NG What is the best approach for the assessment of left atrial size? Comparison of various unidimensional and Two-dimensional parameters with three-dimensional echocardiographically determined left atrial volume J Am Soc Echocardiogr 2006 19 1026 1032 16880098 10.1016/j.echo.2006.03.011

[B17] Khankirawatana B Khankirawatana S Porter T How should left atrial size be reported? Comparative assessment with use of multiple echocardiographic methods Am Heart J 2004 147 369 374 14760338 10.1016/j.ahj.2003.03.001

[B18] Wang Y Gutman JM Heilbron D Wahr D Schiller NB Atrial volume in a normal adult population by two-dimensional echocardiography Chest 1984 86 595 601 6236959 10.1378/chest.86.4.595

[B19] Lang RM Bierig M Devereux RB Flachskampf FA Foster E Pellikka PA Picard MH Roman MJ Seward J Shanewise J Solomon S Spencer KT Sutton MSJ Stewart W Recommendations for chamber quantification Eur J Echocardiogr 2006 7 79 108 16458610 10.1016/j.euje.2005.12.014

[B20] Zabalgoitia M Halperin JL Pearce LA Blackshear JL Asinger RW Hart RG Stroke Prevention in Atrial Fibrillation III Investigators. Transesophageal echocardiographic correlates of clinical risk of thromboembolism in nonvalvular atrial fibrillation J Am Coll Cardiol 1998 31 1622 1626 9626843 10.1016/s0735-1097(98)00146-6

[B21] Leung DY Black IW Cranney GB Hopkins AP Walsh WF Prognostic implications of left atrial spontaneous echo contrast in nonvalvular atrial fibrillation J Am Coll Cardiol 1994 24 755 762 8077549 10.1016/0735-1097(94)90025-6

[B22] Albers GW Dalen JE Laupacis A Manning WJ Petersen P Singer DE Antithrombotic therapy in atrial fibrillation. The Sixth ACCP Conference on Antithrombotic and Thrombolytic Therapy Chest 2001 119 194S 206S 11157649 10.1378/chest.119.1_suppl.194s

[B23] Lip GYH Lane D van Walraven C Hart RG Additive role of plasma von Willebrand Factor levels to clinical factors for risk stratification in patients with atrial fibrillation Stroke 2006 37 2294 2300 16888271 10.1161/01.STR.0000236840.00467.84

[B24] The Stroke Prevention in Atrial Fibrillation Investigators Committee on Echocardiography Transesophageal echocardiographic correlates of thromboembolism in high-risk patients with nonvalvular atrial fibrillation Ann Intern Med 1998 128 639 647 9537937 10.7326/0003-4819-128-8-199804150-00005

[B25] Providência R Trigo J Paiva L Barra S The role of echocardiography in thromboembolic risk assessment of patients with nonvalvular atrial fibrillation J Am Soc Echocardiogr 2013 26 801 812 23791115 10.1016/j.echo.2013.05.010

[B26] Gage BF Waterman AD Shannon W Boechler M Rich MW Radford MJ Validation of clinical classification schemes for predicting stroke: results from the National Registry of atrial fibrillation JAMA 2001 285 2864 2870 11401607 10.1001/jama.285.22.2864

[B27] Lip GY Nieuwlaat R Pisters R Lane DA Crijns HJ Refining clinical risk stratification for predicting stroke and thromboembolism in atrial fibrillation using a novel risk factor-based approach: the euro heart survey on atrial fibrillation Chest 2010 137 263 272 19762550 10.1378/chest.09-1584

[B28] Beppu S Park YD Sakakibara H Nagata S Nimura Y Clinical features of intracardiac thrombosis based on echocardiographic observation Jpn Cite J 1984 48 75 82 10.1253/jcj.48.75 6694334

[B29] Beppu S Nimura Y Sakakihara H Nagata S Park YD Izumi S Smoke-like echo in the left atrial cavity in mitral valve disease: its features and significance J Am Coll Cardiol 1985 6 744 749 4031288 10.1016/s0735-1097(85)80476-9

[B30] Fatkin D Kelly RP Feneley MP Relations between left atrial appendage blood flow velocity, spontaneous echocardiographic contrast and thromboembolic risk in vivo J Am Coll Cardiol 1994 23 961 969 8106703 10.1016/0735-1097(94)90644-0

[B31] Camm AJ Kirchhof P Lip GY Schotten U Savelieva I Ernst S Gelder ICV Al-Attar N Hindricks G Prendergast B Heidbuchel H Alfieri O Angelini A Atar D Colonna P De Caterina R De Sutter J Goette A Gorenek B Heldal M Hohloser SH Kolh P Le Heuzey JY Ponikowski P Rutten FH Guidelines for the management of atrial fibrillation: the task force for the management of atrial fibrillation of the European Society of Cardiology (ESC) Eur Heart J 2010 31 2369 2429 20802247 10.1093/eurheartj/ehq278

[B32] Wann LS Curtis AB Ellenbogen KA Estes M Ezekowitz MD Jackman WM January CT Lowe JE Page RL Slotwiner DJ Stevenson WG Tracy CM 2011 ACCF/AHA/HRS focused update on the management of patients with atrial fibrillation (update on dabigatran): a report of the American College of Cardiology Foundation/American Heart Association Task Force on Practice Guidelines Circulation 2011 123 1144 115021321155

[B33] Ayirala S Kumar S O’Sullivan DM Silverman DI Echocardiographic predictors of left atrial appendage thrombus formation J Am Soc Echocardiogr 2011 24 499 505 21440414 10.1016/j.echo.2011.02.010

[B34] Providência R Botelho A Trigo J Quintal N Nascimento J Mota P Leitão-Marques A Possible refinement of clinical thromboembolism assessment in patients with atrial fibrillation using echocardiographic parameters Europace 2012 14 36 45 21868410 10.1093/europace/eur272

